# Could Greater Physical Activity Reduce Population Prevalence and Socioeconomic Inequalities in Children’s Mental Health Problems? A Policy Simulation

**DOI:** 10.1097/EDE.0000000000001113

**Published:** 2019-12-02

**Authors:** Sungano Chigogora, Anna Pearce, Catherine Law, Russell Viner, Catherine Chittleborough, Lucy J. Griffiths, Steven Hope

**Affiliations:** From the a Population, Policy and Practice Programme, Great Ormond Street Institute of Child Health, University College London, London, United Kingdom; b MRC/CSO Social and Public Health Sciences Unit, University of Glasgow, Glasgow, United Kingdom; c School of Public Health, Robinson Research Institute, The University of Adelaide, Adelaide Health and Medical Sciences Building, South Australia, Australia; d Health Data Research UK, Wales and Northern Ireland, Swansea University Medical School, Wales, United Kingdom.

**Keywords:** Anxiety, Child, Depression, Health inequalities, Mental health, Physical activity

## Abstract

One in four children 5–16 years (y) of age shows signs of mental health problems in the United Kingdom; risk is higher in economically disadvantaged groups. Greater physical activity is associated with lower risk of internalizing problems such as depression and anxiety. We simulated the potential impact of population-wide physical activity interventions on overall prevalence of internalizing problems, and by family income. Interventions were based on the World Health Organization (WHO) children’s target of 60 minutes (min) of moderate-to-vigorous physical activity per day and trial evidence.

**Methods:**

Data were from the UK Millennium Cohort Study, a population-representative cohort of children born in 2000–2002. Household income (5 y) was the exposure; internalizing problems (outcome) were measured using the Strengths and Difficulties Questionnaire (11 y). Of 18,296 singletons, 6,497 had accelerometer physical activity data (mediator, manipulated to simulate interventions) at 7 y. We predicted probabilities of outcome according to exposure in marginal structural models, weighted for attrition and confounding, and adjusted for observed mediator. We then re-estimated probabilities in different physical activity intervention scenarios, assessing income inequalities in internalizing problems with risk ratios (RRs) and differences (RDs) according to income quintile.

**Results:**

Simulating universal achievement of the WHO target led to little change in prevalence (10% [95% CI = 8%, 12%]) and socioeconomic inequalities in internalizing problems; RR: 2.2 (1.1, 3.4); RD: 8% [5%,13%]). More modest increases in physical activity achieved weaker results.

**Conclusions:**

Our simulations suggest that large increases in moderate-to-vigorous physical activity in the United Kingdom would have little effect on prevalence and inequalities in child mental health problems.

## Abstract 2

Supplemental Digital Content is available in the text.

One in four children 5–16 years (y) of age shows signs of mental health problems in the United Kingdom, with 4% clinically diagnosed with an internalizing disorder such as anxiety or depression.^1^ Children from poorer socioeconomic backgrounds are at greater risk of mental health problems.^2^ Internalizing disorders can disrupt learning and behavior, and can cause biologic changes (such as prolonged elevation of stress hormones and abnormal brain development), leading to unhealthy lifestyles, chronic diseases, and widening inequality.^3^ Identifying measures that may support reduction in prevalence and socioeconomic inequalities in internalizing child mental health problems is a policy priority.^4^

Regular exercise is associated with better mental health in children.^5,6^ In observational studies, young people (3–21 y of age) who engage in greater levels of physical activity often report fewer signs of anxiety or depression (such as emotional and peer problems).^5^ Furthermore, randomized trial findings show that increased moderate (e.g., brisk walking) to vigorous (e.g., running) physical activity^7^ can result in reductions in reported internalizing problems.^6^ However, higher levels of physical activity are also associated with more externalizing mental health problems, where children with externalizing problems are more physically active.^5^ The contradictory associations observed between physical activity and child mental health problems may be due to the overlap between physical activity and measures of externalizing disorders (hyperactivity and conduct problems).^8^

Guidance from the UK Chief Medical Officer, following World Health Organization (WHO) recommendations, suggests that children 5–18 y of age should undertake at least 60 minutes (min) of moderate-to-vigorous physical activity per day to optimize muscular, cardiorespiratory, bone, and functional development.^9,10^ Although physical activity levels are generally low in the United Kingdom (with about half of 7-year-olds achieving the 60 min/day target),^11^ there is contradictory evidence on its social patterning.^12^ Children from less affluent backgrounds are more likely to engage in active commuting to school, largely due to lower levels of car ownership among poorer families, and are more likely to engage in unstructured outdoor play.^12^ In contrast, children in more affluent families are more likely to have access to organized sporting activities, clubs, and recreational facilities.^13^

Although interventions to increase physical activity can reduce internalizing problems, a review concluded that the evidence base for physical activity and child mental health problems is limited and often of poor quality.^5^ Despite the lack of good evidence, physical activity is promoted as having benefits for mental health at all ages.^14^ It is not possible to determine the potential impact of increasing moderate-to-vigorous physical activity on population prevalence and inequalities in internalizing mental health problems from trials, due to limitations of small sample size, and heterogeneous design and evaluation. Population roll-out of physical activity interventions would require consideration of several factors. First, criteria for eligibility should maximize the number of children who would benefit from the intervention in light of constrained resources, while promoting equity. Proportionate universalism, where all children receive some level of intervention but some children are offered more intensive intervention based on certain characteristics, may reduce inequality.^15^ In addition, it is important to consider the proportion of eligible children who may engage with the intervention (uptake) and by how much the intervention would actually increase physical activity in those children (effectiveness). Large-scale population data allow for exploration of the potential impact of moderate-to-vigorous physical activity interventions on internalizing mental health problems, informed by existing intervention evidence and actual or hypothetical policy guidance.^16^

The aim of our study was to examine how population-level interventions to increase moderate-to-vigorous physical activity in childhood might reduce prevalence and inequalities in internalizing mental health problems in the United Kingdom, using nationally representative data from the Millennium Cohort Study. We simulated a number of scenarios where an intervention would be available to everyone (universal), to children at increased risk of mental health problems (targeted), or to those already affected by mental health problems (indicated).^17^ We simulated the effectiveness of each intervention according to physical activity increases needed for universal achievement of the WHO 60 min/day moderate-to-vigorous physical activity target, and according to actual increases in physical activity shown in trials. Modeling of uptake was informed by the proportion of children or families who enrolled in previous physical activity intervention trials, accounting for variation between socioeconomic groups (differential uptake).

## METHODS

### Study Characteristics

The Millennium Cohort Study (MCS) is a prospective cohort study of children born in the United Kingdom in 2000–2002, which was constructed to be representative of the total UK population at baseline.^18^ Data on a range of demographic, behavioral, developmental, health, and parental socioeconomic characteristics were recorded for the first time when the child was approximately 9 months old.^19^ Interviews were carried out by trained interviewers, with information provided by a main caregiver (usually the mother). Information was collected on 18,818 infants; our analyses were limited to 18,296 singletons.^20^ We use data collected at ages 5 (n = 15,041), 7 (n = 13,681), and 11 (n = 13,475) years. Ethical approval for data collection at each sweep of MCS and accelerometer studies was granted through the National Health Service, and Northern and Yorkshire Research Ethics Committee systems,^21^ and approval for seasonal accelerometer and calibration studies was granted by the University College London Research Ethics Committee.^22^

### Measures

#### Exposure: Household Income

We used equivalized household income (quintiles), reported at 5 y, as an indicator of socioeconomic circumstances.^23^ We adopted highest level of maternal academic attainment as an alternative indicator of socioeconomic circumstances in a sensitivity analysis.

#### Mediator: Physical Activity

Physical activity was recorded at 7 y using Actigraph GT1M accelerometers, demonstrated to measure activity reliably in children.^24^ Data were processed to eliminate nonwear time and extreme readings, resulting in a physical activity study sample with valid data of ≥10 hours (h) on at least 2 days.^25^ Missing physical activity data were not imputed. Moderate-to-vigorous physical activity was defined as >2,241 accelerometer counts of activity per minute,^11,22^ and was reported in terms of number of minutes of activity per day. Moderate physical activities are typically those that cause the child’s pulse, body temperature, and respiratory rate to increase, while still being able to carry on a conversation (e.g., bike-riding and playground activities); vigorous activities cause the respiratory rate to increase to a point where they find it difficult to carry on conversation (e.g., fast running and swimming).^10^ Further details on the exact levels of these activities used to classify children in the MCS are provided elsewhere.^11^ A Box–Cox transformed measure of moderate-to-vigorous physical activity (*»* = 0.34) was used in regression models as the variable was not normally distributed.^26^ There was no evidence that the effect of moderate-to-vigorous physical activity on internalizing problems varied by income quintiles (*P* value for effect modification = 0.668).

#### Outcome: Internalizing Child Mental Health Problems

Child mental health problems were assessed at 11 y using the Strengths and Difficulties Questionnaire (SDQ), a widely used measure of child behavior completed by the parent/main caregiver. The SDQ comprises 25 items measuring five scales: emotional symptoms, conduct problems, hyperactivity, peer relationship problems, and prosocial behavior.^27^ We examined internalizing mental health problems, combining emotional and peer problems scales; scores ranged from 0 to 20, which we dichotomized using a cut-off (10% highest scores; range 8–19) to indicate internalizing mental health problems.^28^ As sensitivity analyses, we used teacher-reported internalizing scores,^29^ and an alternative measure of internalizing mental health problems, derived by combining all children who had abnormal or high scores on either the peer (score ≥3) or emotional (score ≥4) subscales, based on SDQ recommended subscale cut-offs.^28^

### Confounding

**FIGURE 1.**
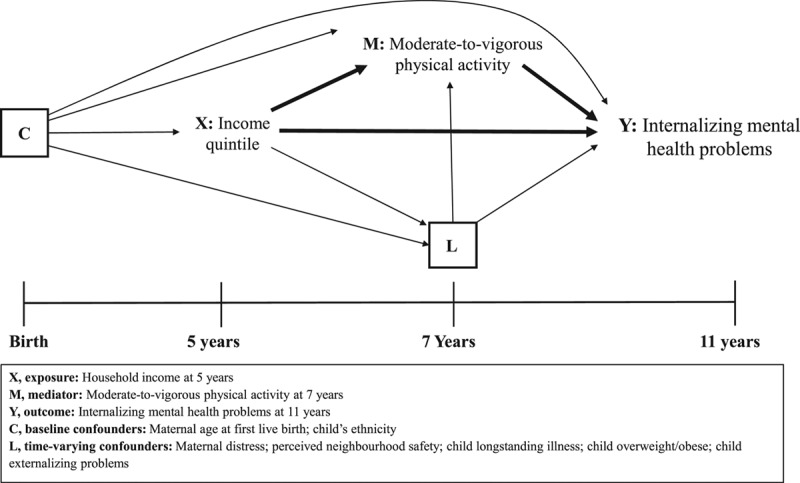
Directed acyclic graph illustrating the relationship between household income (exposure), physical activity (mediator), and internalizing child mental health problems (outcome).

We adjusted for the following factors which were identified as potential confounders, guided by a directed acyclic graph (Figure 1). At baseline, we accounted for maternal age at first live birth and ethnicity. Time-varying covariates were measured at 7 y, the sweep when physical activity was recorded: perceived neighborhood safety, maternal psychological distress (Kessler-6),^30^ child overweight/obese status, and child longstanding illness. To reduce potential for reverse causation by externalizing behavior on physical activity, we accounted for conduct and hyperactivity problems (measured using the SDQ) at 7 y. There was no evidence that inequalities in internalizing mental health problems varied by sex (*P* value for effect modification = 0.754); results are presented for boys and girls combined.

### Intervention Scenarios

We modeled a series of scenarios reflecting varying levels of effectiveness, eligibility, and uptake used in previous simulations of physical activity interventions and childhood overweight. ^16^ Scenarios were guided by the WHO 60 min/day target for moderate-to-vigorous physical activity^10^ and reviews of physical activity interventions.^31–34^

### Effectiveness

To simulate effectiveness, we modified the physical activity variable by increasing number of minutes of activity per day by given amounts, ranging from an average 2.3 min/day (scenario 2) to 30 min/day (scenario 1).

### Eligibility

Each intervention scenario had different eligibility criteria. For example, children were eligible for an active transport intervention dependent on whether their usual mode of travel to and/or from school at 7 y involved walking or cycling, according to maternal report.^33, 34^ Children were eligible for a targeted, after-school intervention if they lived in deprived areas,^32,35^ or an indicated family-based intervention^36^ if they had a borderline/abnormal score for internalizing mental health problems at 5 y.

### Uptake

Uptake levels varied for each scenario, from 100% in a hypothetical intervention where children would increase their physical activity in accordance with the WHO target of 60 min/day, to 64% in the indicated family-based intervention.^36–40^ Table 1 contains a detailed description of each scenario.

**TABLE 1.**
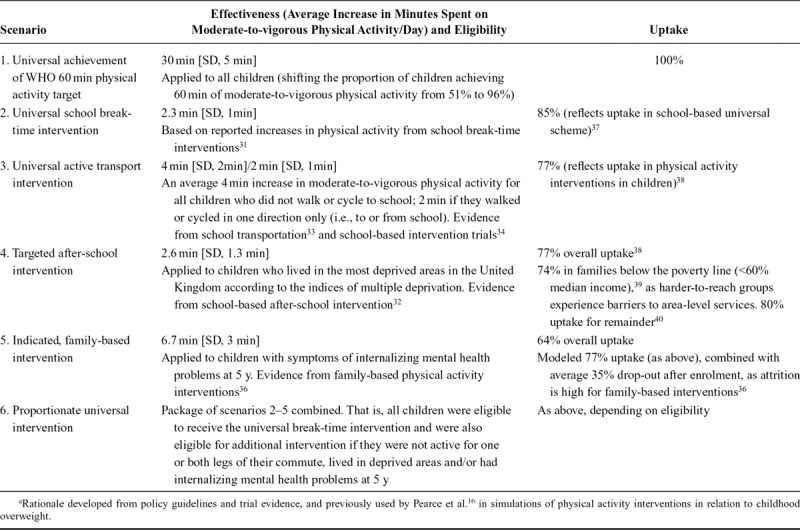
Rationale for Previously Defined Intervention Scenarios and Simulated Effect Sizes^a^

### Statistical Analysis

We used descriptive statistics (proportions, means, and medians with 95% confidence intervals [CIs], weighted for attrition) to illustrate associations between household income, moderate-to-vigorous physical activity, internalizing mental health problems, and covariates.

### Prevalence of Internalizing Problems and Measures of Inequality

We estimated the association between income and internalizing problems using logistic regression, weighted for attrition to the MCS physical activity study and survey design,^22^ with internalizing scores up to the 90th percentile as referent, versus the highest 10%. We used predicted probabilities (and 95% confidence intervals) obtained from this regression model to estimate the prevalence of internalizing problems, overall and in each income quintile. We estimated summary measures of relative and absolute inequalities by repeating the regression with income quintile as a continuous linear term. Relative inequalities were calculated as the ratio of fitted probabilities of internalizing problems between the highest and lowest income quintiles (risk ratio [RR]; 95% confidence interval [CI]), and absolute inequalities as the difference between fitted probabilities for the highest and lowest income quintiles (risk difference [RD] and 95% CI).

### Adjusting for Baseline Confounding Between Household Income and Internalizing Problems

We accounted for baseline confounding in a marginal structural model (MSM) using inverse probability of treatment weights (IPTWs, eAppendix1; http://links.lww.com/EDE/B596).^41^ This provided an estimate of the total direct effect (TDE) of income on internalizing problems. To remove the excessive influence of extreme values, all IPTWs were trimmed at the 1st and 99th percentiles^42^ and multiplied these by the MCS physical activity study weight.^22^

### Adjusting for Baseline and Time-varying Confounding, and the Mediating Role of Moderate-to-vigorous Physical Activity

We entered observed moderate-to-vigorous physical activity in minutes into the MSM, with both baseline and intermediate confounders accounted for using IPTWs. This provided the “observed” controlled direct effect (CDE) of income on internalizing problems, an estimate of the effect of household income on child internalizing mental health problems, adjusted for observed level of physical activity and confounding.

### Simulation of Intervention Scenarios

We simulated the effect of each intervention scenario according to effectiveness, eligibility criteria, and uptake as described above and in Table 1. This was achieved by re-estimating the predicted probabilities of internalizing problems from the observed controlled direct effect model after increasing the physical activity (mediator) value by an amount (in minutes), as set out in the scenario. For example, in scenario 3, a universal transport intervention was simulated. This resulted in increases of physical activity by an average of 4 min (with a standard deviation [SD] of 2 min) in children whose commutes were not active to and from school (involving car, taxi, or bus ride), and by an average 2 min (SD 1min) in children who were not active in one of the two commutes. This intervention was modeled for 77% uptake according to trial evidence. ^37^ The process we followed to manipulate the physical activity variable (as if changed by intervention) has been applied elsewhere,^16,43^ and is described in detail in eAppendix 2; http://links.lww.com/EDE/B596.

All analyses were performed in Stata SE 15.1.

### Working Sample

**FIGURE 2.**
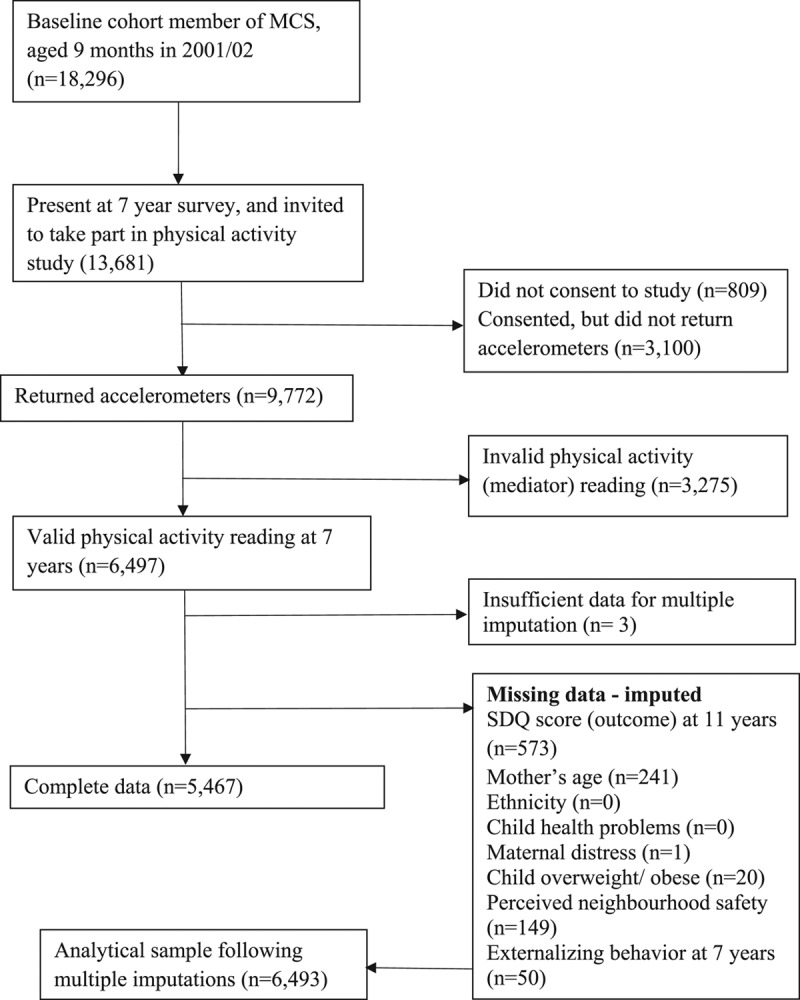
Selection of cohort members for investigation of the role of physical activity in the association between socioeconomic circumstances and mental health, Millennium Cohort Study.

Of 18,296 singletons recruited to the Millennium Cohort Study, 13,681 of participants at 7 y were invited to take part in the accelerometer study. Parental consent was given for 12,872 children, and 9,722 returned the accelerometers at the end of the study. Valid readings were recorded for 6,497 children. In total, 1,467 children had missing outcome or covariate data; these data were imputed under a missing at random assumption for 1,464 children (three had insufficient auxiliary information) (Figure 2). This was done through multiple imputation by chained equations in 20 datasets using auxiliary information, combined using Rubin’s rules.^44^ The augment function was used to ensure that previously empty unrelated cells were unaffected.

### Sensitivity Analyses

We carried out four sensitivity analyses. In the first, we replaced parental reports of internalizing mental health problems (outcome) with those provided by a teacher (eTable 1; http://links.lww.com/EDE/B596), which comprised a much smaller subsample of children who took part in the MCS physical activity study. In the second, we replaced the distribution-based outcome with clinical cut-offs of the two subscales that comprise internalizing mental health problems (eTable 2; http://links.lww.com/EDE/B596).^28^ This is a less stringent cut-off, which includes children who scored high on either the emotional and peer subscale. In the third, we used highest maternal educational attainment as an alternative indicator of socioeconomic circumstances (exposure, eTable 3; http://links.lww.com/EDE/B596). In the fourth, we repeated analyses in children who had values for all covariates (complete cases, eTable 4; http://links.lww.com/EDE/B596).

## RESULTS

### Sample Characteristics

Table 2 shows characteristics of the original MCS sample, the multiply imputed analytic sample, and a complete-case sample. In general, the distribution of variables was consistent across all three samples.

**TABLE 2.**
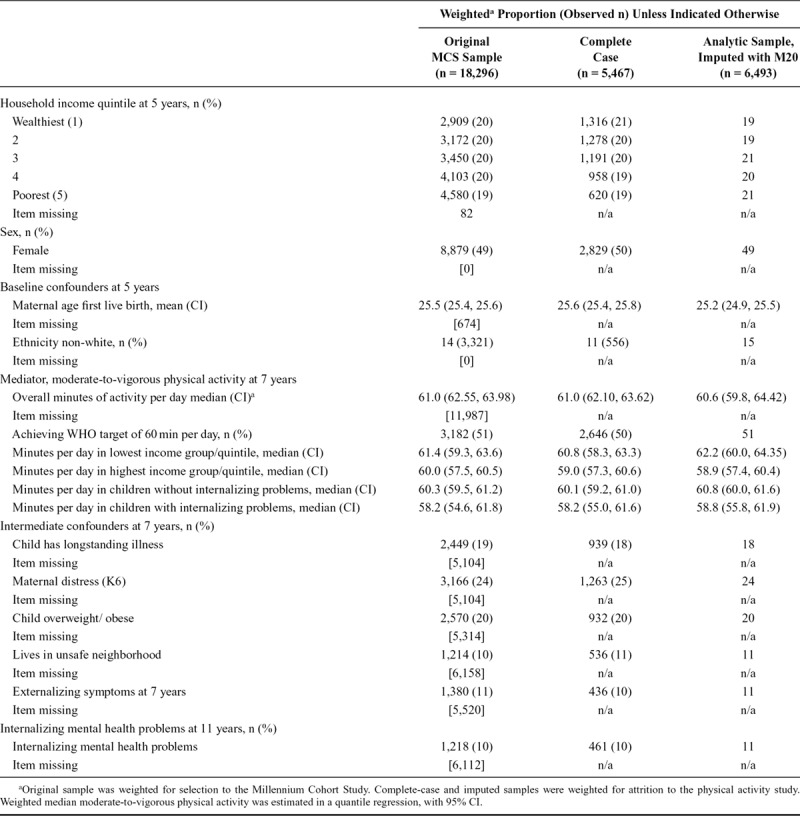
Characteristics of Millennium Cohort Study Members in Original and Analytic Samples

### Descriptive Findings

In the main (imputed) analytic sample, 11% of children had internalizing mental health problems, median moderate-to-vigorous physical activity was 61 min, and 51% of children met the WHO physical activity target of 60 min per day. A weak reverse gradient in physical activity was observed, where children from the lowest household income quintile had greater activity (median 62 min) compared with those in the highest quintile (median 59 min). Median time spent in moderate-to-vigorous physical activity at 7 y was slightly lower in children with internalizing mental health problems (59 min), compared with those without (61 min). The weak reverse gradient in physical activity according to income suggests that increasing physical activity levels may not reduce inequality in internalizing problems.

### Observed Controlled Direct Effect of Household Income on Internalizing Problems

Accounting for attrition to the physical activity study (model A), population prevalence of internalizing mental health problems was 11% (95% CI = 10%, 12%; Table 3). Compared with children in the highest quintile, those from families in the lowest quintile of household income were more likely to have internalizing problems, according to measures of both relative and absolute inequality (RR: 3.3 [2.3, 4.4]; RD: 12% [8.6, 15.4]). Prevalence and inequalities were attenuated after adjustment for baseline confounding (the total direct effect/TDE of income on internalizing problems, model B). Additional adjustment for confounding and observed physical activity (the observed controlled direct effect/CDE, model C) resulted in little change from the TDE (overall prevalence 10% [9, 12]; RR: 2.2 [1.1, 3.4]; RD: 8% [3, 13]).

**TABLE 3.**
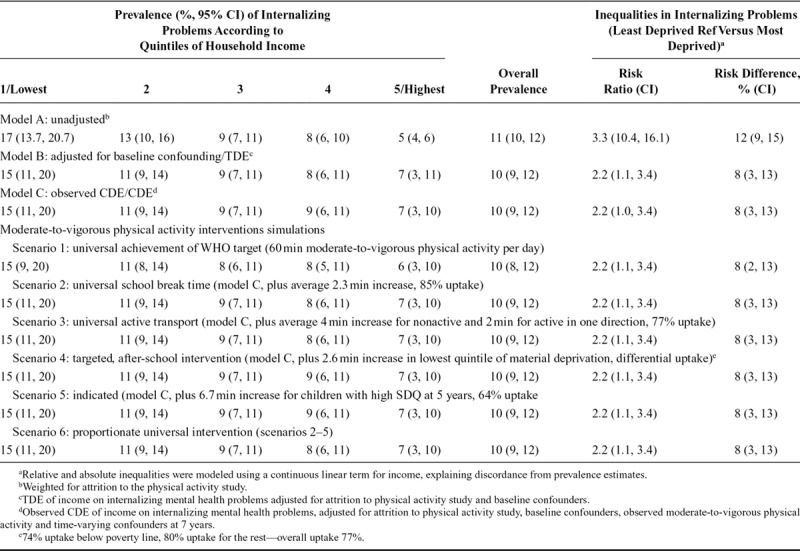
Prevalence and Risk of Parent-reported Internalizing Child Mental Health Problems at 11 Years According to Household Income at 5 Years: Millennium Cohort Study

### Intervention Scenarios

A universal increase in moderate-to-vigorous physical activity by an average of 30 min for each child in the MCS physical activity study (scenario 1) resulted in 95.6% attainment of the WHO recommended 60 min per day (Table 3). Increasing physical activity by this amount slightly reduced overall prevalence of internalizing mental health problems to 10% [8, 12], compared with the observed CDE. Absolute inequality was also lower compared with the observed CDE (RD 8% [2, 13]), but relative inequality remained unchanged.

More realistic increases in physical activity (scenarios 2–5) did not reduce prevalence or inequalities in internalizing problems. The effect of increasing moderate-to-vigorous physical activity was the same whether it was done universally during break time (scenario 2),^31^ on the school-commute (scenario 3), targeted to children living in deprived areas (scenario 4), or applied to children with prior internalizing problems (scenario 5). The combination of universal, targeted, and indicated interventions (scenarios 2–5) within a single package of interventions resulted in only slightly lower prevalence and absolute inequalities than shown for the constituent scenarios in isolation.

### Sensitivity Analyses

We conducted sensitivity analyses using alternative measures of the outcome (eTable 1; http://links.lww.com/EDE/B596 and eTable 2; http://links.lww.com/EDE/B596), and these showed that increasing moderate-to-vigorous physical activity at 7 y would make little difference to later reports of internalizing mental health problems regardless of the rater, and whether less stringent or clinical cut-offs of internalizing problems were applied. Sensitivity analyses with maternal education (eTable 3; http://links.lww.com/EDE/B596) as an alternative measure of the socioeconomic status (the exposure) showed similar results to those found using income as a measure of socioeconomic circumstances. We also conducted analyses in a complete cases sample, with nonmissing covariate data (eTable 4; http://links.lww.com/EDE/B596), which showed that our findings were substantially the same as in the multiply imputed analytic sample.

## DISCUSSION

### Main Findings

A simulation of the impact of increasing moderate-to-vigorous physical activity in children 7 years of age according to the WHO target of 60 min/day resulted in a small reduction in population prevalence of internalizing mental health problems at 11 y. More realistic simulations, informed by physical activity increases and uptake achieved in trials, showed only a small reduction when interventions were used in combination.

Marmot et al.^15^ advocate a proportionate universal approach for improving population health and reducing inequalities. Results from our simulation of proportionate universal interventions, where intensive physical activity interventions were applied to targeted groups in addition to universal implementation, resulted in slightly lower prevalence and absolute inequality in internalizing mental health problems than for individual interventions. However, the reduction observed was so small that it would not justify the extra resources required to implement such a combined approach.

### Comparison with Other Studies

Our results support findings from other studies showing that children from less advantaged backgrounds are at greater risk of child mental health problems.^2^ Studies examining physical activity by socioeconomic circumstances have produced contradictory results.^12^ Our finding of a weak reverse gradient between physical activity and household income corroborate previous findings from the Millennium Cohort Study, which demonstrated that disadvantaged children were more likely to be physically active.^11,45^

Observational studies and randomized trials have shown beneficial effects of physical activity on child mental health problems, where greater physical activity was associated with lower levels of internalizing problems.^5^ However, it has been difficult to synthesize evidence from physical activity trials to inform interventions at the population level, due to variation in methodologic characteristics such as sampling and measurement. Using an alternative approach, we have combined physical activity interventions shown to be effective in children and estimated their potential impact on internalizing mental health problems in a population-representative sample. Our findings indicate that increasing the physical activity of children through a proportionate universal approach, consisting of several evidenced interventions, would have little effect on prevalence and inequality in internalizing mental health problems in the United Kingdom.

### Strengths and Limitations

This is the first study to use intervention simulations to examine how increasing moderate-to-vigorous physical activity in the general population could affect child mental health problems. We analyzed data from a large, nationally representative cohort of UK children with an objective measure of physical activity and rich socioeconomic, health, and demographic data, which we used to operationalize targeting mechanisms and account for potential confounders. The availability of longitudinal data allowed for temporal ordering of household income and internalizing mental health problems. We used subscales of the Strengths and Difficulties Questionnaire, a comprehensive, validated, and widely used measure of child mental health problems, using both distribution and clinical cut-offs.

Findings from our study were largely the same in sensitivity analyses according to different raters of internalizing problems, alternative measures of socioeconomic circumstances, and restriction to a complete-case sample.

We were able to examine a number of population intervention scenarios. The eligibility, uptake, and effectiveness of the policy scenarios were carried out in earlier work and guided by the WHO physical activity target, evidence from physical activity trials, systematic reviews of physical activity studies, and consultation with experts and parents.^16^ However, trial evidence of the relationship between physical activity and internalizing mental health problems was scarce, so intervention scenarios were informed by physical activity interventions designed to improve other outcomes (such as overweight)^16^; nevertheless, this still provides an informative picture of increases in physical activity that might realistically be achieved through intervention. Additional details about the effectiveness of physical activity interventions according to the socioeconomic circumstances of recruited children would have helped to more accurately model increases in physical activity. With more nuanced trial evidence, we could also have modeled alternative intervention scenarios showing how uptake of physical activity interventions may differ across socioeconomic groups. Trials where physical activity interventions were combined with other lifestyle characteristics, such as diet, could have been used to inform scenarios reflecting the complex environment where multiple risk factors co-exist. While we were able to examine a hypothetical proportionate universal intervention, where we combined a number of interventions (scenarios 2–5), such a physical activity intervention may not be practical in real life, where families and children may be too busy to engage with every intervention on offer to them. Thus, it is possible that the small effects we modeled would not be observed in the general population.

Our analyses were somewhat limited by data availability. First, we did not have information about distance between homes and schools and any barriers to active commuting (e.g., busy roads or lack of sidewalks), which we could have used to inform our active transport intervention scenario. Second, although physical activity was objectively measured in our study, some measurements were removed due to extreme values and time not wearing accelerometers, thus excluding some children from our investigation.^22^ Third, although we adjusted for a number of baseline and intermediate confounding factors, there may be residual confounding that was not measured.

### Implications and Future Research

Following WHO recommendations that all children should achieve 60 min of moderate-to-vigorous physical activity per day,^10^ a national target has been set to reach this goal in the United Kingdom.^9^ Our analysis indicates that if this target were to be achieved, the impact on prevalence and inequalities in child mental health problems would be small. Efforts to increase moderate-to-vigorous physical activity beyond this may be unrealistic in light of evidence that at least half of children 7 y of age were not achieving that target in the MCS.^11^

Our analyses demonstrate a method for simulating population interventions according to universal or targeted eligibility criteria, with differing levels of effectiveness and uptake. The simulations allowed us to ask questions relevant to policy on child physical activity and mental health, addressing knowledge gaps that cannot be addressed through trial evidence. A similar approach could be adopted to evaluate potential impacts of the WHO 60 min/day moderate-to-vigorous physical activity target for other outcomes, and in other nations. This approach could also help anticipate the consequences of other policies or targets being considered by policy-makers before they are implemented, and to address other relevant questions about the potential real-world impact of policy interventions.

Although promotion of physical activity may benefit child mental health, the effects of the interventions we modeled were small. One plausible explanation for the small effects of moderate-to-vigorous physical activity on internalizing mental health problems may be the prolonged interval between physical activity measurement at 7 y and internalizing mental health problems at 11 y. Trial evidence has generally reported short follow-up periods, within a few weeks of intervention,^5^ and our findings may possibly reflect the reduced impact of physical activity interventions over a longer duration. The lack of evidence of notable long-term effects of moderate-to-vigorous physical activity on internalizing child mental health problems suggests the need for trials with longer follow-up to assess lasting effects of physical activity on child mental health problems. In addition, researchers investigating the association between physical activity and child mental health problems should consider the impact of measurement of mental health problems, specifically the potential complex interrelationships between moderate-to-vigorous physical activity and externalizing behaviors, which are commonly incorporated in measures of child mental health.

Our study suggests that increasing physical activity in children in the United Kingdom according to WHO policy guidance may have little effect on prevalence and inequality in internalizing mental health problems. Future research should examine how physical activity interventions could benefit inequalities in other childhood outcomes known to be associated with physical activity, such as muscular, cardiorespiratory, bone and functional development.^9^

## Supplementary Material
