# Supplementary material for: Could Greater Physical Activity Reduce Population Prevalence and Socioeconomic Inequalities in Children’s Mental Health Problems? A Policy Simulation
Source: Epidemiology. 2019 Dec 2;31(1):115–25. doi: 10.1097/EDE.0000000000001113 (PMC6889907; doi:10.1097/EDE.0000000000001113)
Supplement: Supplementary file 1 [file ede-31-115-s001.docx]

**eAppendix 1: Inverse probability of treatment weights**

This supplement illustrates how we computed inverse of probability weights to account for confounding, before we simulated the effects of possible physical activity interventions. Intervention simulations were applied in the same way by Pearce et al (2018)^16^ and Chittleborough et al (2014),^43^ who are cited in the manuscript.

Analyses were performed in Stata SE 15.1.

The inverse probability of treatment weight applied in the controlled direct effect/ CDE model (IPTW)^41^ consisted of three weights multiplied. These three weights represented:

*Weight 1: Attrition to the Millennium Cohort Physical Activity Study and sampling design.*

This weight (DOVWT2PA in dataset), was derived by the Centre for Longitudinal Study and supplied in Millennium Cohort Study dataset. ^22^

*Weight 2: Baseline confounding*

Change in probability of income quintile before and after adjustment for baseline confounders (maternal age at first live birth, ethnicity)

*Weight 3: Time-varying confounding*

Change in probability density function (assumed normal) of moderate-to vigorous physical activity given income quintile, before and after adjustment for baseline confounders (maternal age at first live birth, ethnicity) and intermediate confounders (perceived neighborhood safety, maternal psychological distress (Kessler-6), child overweight/obese status, child longstanding illness and conduct/ hyperactivity problems at 7y).

*Final weight for controlled direct effect/ CDE model*

Weight 1 X Weight 2 X Weight 3

Weights 1 and 2 were computed in an imputed dataset (using mi_ commands) as following steps

1. Generate variables for weights
2. Predict probability of *the exposure* (numerator) [for Weight 2]
3. Predict probability of *the exposure*, given baseline confounders, C1 and C2 (denominator), [for Weight 2]
4. Stabilize *Weight 2* by dividing the numerator by the denominator.
5. Predict probability of *the mediator*, given the exposure (numerator2) [for Weight 3]
6. Predict probability of *the mediator,* given the exposure, baseline and intermediate confounders, C1 and C2 (denominator2), [for Weight 3]
7. Stabilize *Weight 2* by *dividing numerator2 by denominator2*
8. Truncate Weight 2 and Weight 2
9. Multiply *Weight1* X *Weight* 2 X *Weight 3*

Values below the 1^st^ centile and above the 99^th^ were recoded as the values at the 1^st^ and 99^th^ centiles. This trimming procedure is recommended in order to avoid observations with extremely large weights, as these may unduly influence results and produce estimates with high variance.^42^ Figures S1 and S2 contain boxplots for the ITPW before and after these procedures.

***Figure S1: Boxplot of the untrimmed IPTW (w) (mean = 1.0 [range 0.2-9.9])***

**

***Figure S2: Boxplot of the trimmed IPTW (w99) (mean = 0.98 [range 0.30-1.6])***

**eAppendix 2: How the physical activity variable was manipulated**

This supplement illustrates how we manipulated the observed physical activity variable in each of our scenarios, as if changed by intervention. Similar methods have been applied elsewhere.^16, 43^

The observed physical activity variable (*O_d_*) consisted of the average number of minutes a child spent engaging in moderate-to-vigorous physical activity each day. A new physical activity variable (*N_d_*) was created, initially taking on the values of the observed physical activity variable (*O_d_*). *N_d_* is then manipulated, as if changed by an intervention with effectiveness *Ef,* eligibility *El* and uptake *U*

*Ef* represents *the average* effectiveness (or increase in physical activity), informed by the relevant intervention evidence (Table 1). *Ef* minutes is an *average* increase in physical activity, as there would be some variability in the amount of improvement in an actual intervention. Therefore, *N_d_* is increased, for each child, by an amount drawn from a distribution which is assumed to be normal, with mean *Ef* and standard deviation *SD*. The *SD* is not determined by the intervention evidence, but is an arbitrary figure chosen to provide a realistic amount of change that might be expected from an intervention, while also preserving the distribution of *O_d_.*

*El* represents eligibility for the intervention and is relevant for scenarios where the simulated intervention was not universal. A variable was created to represent *El* and an increase of *Ef* was only applied to children with *El*=1.

*U* represents % uptake and allowed for the fact that not all children who are eligible for an intervention will receive it. An increase of *Ef* (SD) is therefore applied to *U*% of *El* children. We assumed that uptake, within eligible groups, was random. For scenarios where U<100%, a new eligibility variable (*ElU*) was created, which randomly assigned *ElU* status to *U*% of *El* children.

***Using Scenario 4 as an example:***

Targeted, after school intervention providing additional 2.6 minutes activity for children identified as living in the most deprived areas in the UK according to indices of multiple deprivation. Children who lived in deprived neighbourhoods at age 5y were classified as El=1 (i.e. *eligible* for the intervention). A randomly drawn 77% of *EI*=1 (living in deprived neighbourhoods) children were classified as *ElU­*=1 (allowing for *uptake*). *ElU­*=1 children (i.e. among 77% of those who lived in deprived neighbourhoods at age 5) were assigned an increase in physical activity that was drawn from a normal distribution with mean 2.6, SD 1.

*Applying Scenario 4, prefixed by mi_ commands in Stata SE 15.1*

1. Compute the probability of the outcome given exposure and observed physical activity in minutes. Adjust for attrition, baseline and intermediate confounding using the combined weight described in supplement 1.
2. Generate a physical activity variable for scenario 5, replacing it with the observed physical activity variable
3. Compute *post-intervention* physical activity values: (observed+ *rnormal*(2.6,1. 3), if *ElU­*=1
4. Predict the probability of the outcome as in controlled direct effect/ CDE model
5. Replace the observed physical activity value with the post-intervention values computed in step 3
6. Estimate overall post-intervention prevalence of the outcome, using the *mimrgns* command
7. Estimate prevalence in each exposure group
8. Expressing the exposure as a continuous variable, estimate the Risk Ratio and Risk Difference using the *nlcom* command

**eTable 1: Prevalence and risk of teacher reported internalizing problems at age 11 years according to household income at 5 years: Millennium Cohort Study**

| **Prevalence (%, 95% confidence interval [CI]) of internalizing problems according to quintiles of household income** | | | | | **Overall prevalence** | **Inequalities in internalizing problems (least deprived ref v most deprived)^d^** | |
| --- | --- | --- | --- | --- | --- | --- | --- |
| 1/lowest | 2 | 3 | 4 | 5/highest |  | Risk ratio (CI) | Risk difference, % (CI) |
| **Model A:** Unadjusted^a^ | | | | | | | |
| 17 (12, 22) | 15 (12, 18) | 13 (10, 16) | 10 (7, 12) | 8 (6,10) | 12 (11, 15) | 2.0 (1.4, 2.8) | 9 (5, 14) |
| **Model B:** Adjusted for baseline confounding/ TDE^b^ | | | | | | | |
| 17 (11, 23) | 1511, 18) | 13 (10, 16) | 10 (7, 13) | 9 (5, 14) | 13 (11, 15) | 1.9 (1.0, 2.7) | 8 (2, 14) |
| **Model C:** Observed controlled direct effect/ CDE^c^ | | | | | | | |
| 17 (11, 23) | 15 (11, 18) | 13 (10, 15) | 10 (7, 13) | 9 (5, 14) | 13 (11, 15) | 1.9 (1.0, 2.8) | 8 (2, 14) |
| **Moderate-to-vigorous physical activity interventions simulations** | | | | | | | |
| **Scenario 1: Universal achievement of WHO target (**60min moderate-to-vigorous physical activity per day) | | | | | | | |
| 16 (9, 23 | 14 (9, 18) | 12 (8, 15) | 9 (6, 12) | 10 (5, 13) | 12 (9, 15) | 1.9 (1.0, 2.8) | 7.6 (1, 14) |
| **Scenario 2: Universal, school break time** (Model C/ CDE, plus average 2.3min increase, 85% uptake | | | | | | | |
| 17 (11, 23) | 15 (11, 18) | 13 (9, 15) | 10 (7, 13) | 9 (5, 13) | 13 (11, 15) | 1.9 (1.0, 2.8) | 8 (2, 14) |
| **Scenario 3: Universal, active transport** Model C/ CDE, plus average 4min increase for non-active and 2min for active in one direction, 77% uptake | | | | | | | |
| 17 (11, 23) | 15 (11, 18) | 12 (9, 15) | 10 (7, 13) | 9 (5, 13) | 13 (11, 15) | 1.9 (1.0, 2.8) | 8 (2, 14) |
| **Scenario 4: Targeted after school intervention** (Model C/ CDE, plus 2.6 increase in lowest quintile of material deprivation, differential uptake^e^ | | | | | | | |
| 17 (11, 23) | 15 (11, 18) | 12 (10, 15) | 10 (7, 13) | 9 (5, 14) | 13 (11, 15) | 1.9 (1.0, 2.8) | 8 (2, 14) |
| **Scenario 5: Indicated** (Model C/ CDE, plus 6.7 min increase for children with high SDQ at 5 years, 64% uptake | | | | | | | |
| 17 (11, 23) | 15 (11, 18) | 12 (10, 15) | 10 (7, 13) | 9 (5, 14) | 13 (11, 15) | 1.9 (1.0, 2.8) | 8 (2, 14) |
| **SCENARIO 6: Proportionate universal intervention (scenarios 2-5)** | | | | | | | |
| 17 (11, 23) | 14 (11, 18) | 12 (10, 15) | 10 (7, 13) | 9 (5, 13) | 13 (11, 15) | 1.9 (1.0, 2.8) | 8 (2, 14) |

^a^ Weighted for attrition to the physical activity study

^b^ Total direct effect (TDE) of income on internalizing mental health problems adjusted for attrition to physical activity study and baseline confounders

^c^ Observed controlled direct effect (CDE) of income on internalizing mental health problems, adjusted for attrition to physical activity study, baseline confounders, observed moderate-to-vigorous physical activity and time-varying confounders at 7 years

^d^ Relative and absolute inequalities were modelled using a continuous linear term for income, explaining discordance from prevalence estimates

^e^74% uptake below poverty line, 80% uptake for the rest – overall uptake 77%)

**eTable 2: Prevalence and risk of whether child has borderline or abnormal peer or emotional scores at validated SDQ cut-offs at age 11 years according to household income at 5 years: Millennium Cohort Study**

| **Prevalence (%, 95% confidence interval [CI]) of peer or emotional problems according to quintiles of household income** | | | | | **Overall prevalence** | **Inequalities in internalizing problems (least deprived ref v most deprived)^d^** | |
| --- | --- | --- | --- | --- | --- | --- | --- |
| 1/lowest | 2 | 3 | 4 | 5/highest |  | Risk ratio (CI) | Risk difference, % (CI) |
| **Model A:** Unadjusted^a^ | | | | | | | |
| 39 (34, 43) | 31 (27, 35) | 27 (24, 31) | 24 (21, 27) | 18 (16, 21) | 28 (38, 20) | 2.0 (1.7, 2.4) | 19 (15, 24) |
| **Model B:** Adjusted for baseline confounding/ TDE^b^ | | | | | | | |
| 35 (30, 41) | 30 (26, 33) | 28 (25, 31) | 24 (21, 28) | 21 (16, 26) | 28 (26, 29) | 1.6 (1.3, 2.0) | 13 (7, 19) |
| **Model C:** Observed controlled direct effect/ CDE^c^ | | | | | | | |
| 35 (29, 40) | 29 (26, 33) | 28 (25, 32) | 24 (21, 27) | 21 (17, 26) | 28 (26, 30) | 1.6 (1.2, 2.0) | 13 (7, 19) |
| **Moderate-to-vigorous physical activity interventions simulations** | | | | | | | |
| **Scenario 1: Universal achievement of WHO target (**60min moderate-to-vigorous physical activity per day) | | | | | | | |
| 33 (26, 39) | 28 (23, 32) | 26 (22, 31) | 22 (18, 26) | 20 (15, 25) | 26 (23, 29) | 1.6 (1.2, 2.0) | 12 (6, 18) |
| **Scenario 2: Universal, school break time** (Model C/ CDE, plus average 2.3min increase, 85% uptake | | | | | | | |
| 34 (29, 40) | 29 (26, 32) | 28 (24, 31) | 24 (21, 27) | 21 (17, 26) | 27 (26, 29) | 1.6 (1.2, 2.0) | 13 (6, 19) |
| **Scenario 3: Universal, active transport** Model C/ CDE, plus average 4min increase for non-active and 2min for active in one direction, 77% uptake | | | | | | | |
| 35 (29, 40) | 29 (25, 33) | 28 (25, 32) | 24 (21, 27) | 21 (17, 26) | 27 (25, 29) | 1.6 (1.2, 2.0) | 13 (6, 19) |
| **Scenario 4: Targeted after school intervention** (Model C/ CDE, plus 2.6min increase in lowest quintile of material deprivation, differential uptake^e^ | | | | | | | |
| 35 (29, 40) | 29 (25, 33) | 28 (25, 32) | 24 (21, 27) | 21 (17, 26) | 28 (26, 30) | 1.6 (1.2, 2.0) | 13 (6, 19) |
| **Scenario 5: Indicated** (Model C/ CDE, plus 6.7 min increase for children with high SDQ at 5 years, 64% uptake | | | | | | | |
| 35 (29, 40) | 29 (25, 33) | 28 (25, 32) | 24 (21, 27) | 21 (17, 26) | 28 (26, 30) | 1.6 (1.2, 2.0) | 13 (6, 19) |
| **Scenario 6: Proportionate universal intervention (scenarios 2-5)** | | | | | | | |
| 34 (28, 40) | 29 (25, 33) | 28 (24, 31) | 24 (20, 27) | 21 (16, 26) | 27 (25, 29) | 1.6 (1.2, 2.0) | 13 (6, 19) |

^a^ Weighted for attrition to the physical activity study

^b^ Total direct effect (TDE) of income on internalizing mental health problems adjusted for attrition to physical activity study and baseline confounders

^c^ Observed controlled direct effect (CDE) of income on internalizing mental health problems, adjusted for attrition to physical activity study, baseline confounders, observed moderate-to-vigorous physical activity and time-varying confounders at 7 years

^d^ Relative and absolute inequalities were modelled using a continuous linear term for income, explaining discordance from prevalence estimates

^e^74% uptake below poverty line, 80% uptake for the rest – overall uptake 77%)

**eTable 3: Prevalence and risk of parent- reported internalizing problems at age 11 years according to highest level of maternal education: Millennium Cohort Study**

| **Prevalence (%, 95% confidence interval [CI]) of internalizing problems according to maternal education** | | | | | **Overall prevalence** | **Inequalities in internalizing problems (least deprived ref v most deprived)^d^** | |
| --- | --- | --- | --- | --- | --- | --- | --- |
| No qualification | Completed GCSE | A/ AS/ S level | Diploma | Degree and above |  | Risk ratio (CI) | Risk difference, % (CI) |
| **Model A:** Unadjusted^a^ | | | | | | | |
| 16 (12, 20) | 12 (10, 13) | 8 (5, 11) | 5 (3, 8) | 6 (4, 7) | 11 (10, 12) | 3.2 (2.1, 4.3) | 11 (7, 14) |
| **Model B:** Adjusted for baseline confounders/ TDE^b^ | | | | | | | |
| 14 (10, 19) | 11 (9, 13) | 9 (5, 13) | 5 (3, 7) | 5 (4, 7) | 10 (9, 11) | 3.0 (1.8, 4.3) | 10 (6, 13) |
| **Model C:** Observed controlled direct effect/ CDE^c^ | | | | | | | |
| 14 (10, 18) | 12 (9, 14) | 9 (5, 13) | 5 (3, 7) | 5 (4, 7) | 10 (9, 12) | 3.0 (1.7, 4.2) | 10 (6, 13) |
| **Moderate-to-vigorous physical activity interventions simulations** | | | | | | | |
| **Scenario 1: Universal achievement of WHO target (**60min moderate-to-vigorous physical activity per day) | | | | | | | |
| 13 (19, 18) | 11 (8, 14) | 8 (4, 12) | 5 (2, 7) | 5 (3, 7) | 10 (8, 12) | 3.0 (1.7, 4.3) | 9 (5, 13) |
| **Scenario 2: Universal, school break time** (Model C/ CDE, plus average 2.3min increase, 85% uptake | | | | | | | |
| 14 (10, 18) | 12 (9, 14) | 9 (5, 13) | 5 (3, 7) | 5 (4, 7) | 10 (9, 12) | 3.0 (1.7, 4.2) | 10 (6, 13) |
| **Scenario 3: Universal, active transport** (Model C/ CDE, plus average 4min increase for non-active and 2min for active in one direction, 77% uptake | | | | | | | |
| 14 (10, 18) | 12 (9, 14) | 9 (5, 13) | 5 (3, 7) | 5 (4, 7) | 10 (9, 12) | 3.0 (1.7, 4.2) | 10 (6, 13) |
| **Scenario 4: Targeted, after school** (Model C/ CDE, plus 2.6min increase in lowest quintile of material deprivation, differential uptake^e^ | | | | | | | |
| 14 (10, 18) | 12 (9, 14) | 9 (5, 13) | 5 (3, 7) | 5 (4, 7) | 10 (9, 12) | 3.0 (1.7, 4.2) | 10 (6, 13) |
| **Scenario 5: Indicated** (Model C/ CDE, plus 6.7 min increase for children with high SDQ at 5 years, 64% uptake | | | | | | | |
| 14 (10, 18) | 12 (9, 14) | 9 (5, 13) | 5 (3, 7) | 5 (4, 7) | 10 (9, 12) | 3.0 (1.7, 4.2) | 10 (6, 13) |
| **Scenario 6: Proportionate universal intervention (scenarios 2-5)** | | | | | | | |
| 14 (10, 18) | 11 (9, 14) | 9 (5, 13) | 5 (3, 7) | 5 (4, 7) | 10 (9, 12) | 3.0 (1.7, 4.2) | 10 (6, 13) |

^a^ Weighted for attrition to the physical activity study

^b^ Total direct effect (TDE) of income on internalizing mental health problems adjusted for attrition to physical activity study and baseline confounders

^c^ Observed controlled direct effect (CDE) of income on internalizing mental health problems, adjusted for attrition to physical activity study, baseline confounders, observed moderate-to-vigorous physical activity and time-varying confounders at 7 years

^d^ Relative and absolute inequalities were modelled using a continuous linear term for income, explaining discordance from prevalence estimates

^e^74% uptake below poverty line, 80% uptake for the rest – overall uptake 77%)

**eTable 4: Prevalence and risk of parent- reported internalizing problems at age 11 years according to household income at 5 years: Millennium Cohort Study (complete cases)**

| **Prevalence (%, 95% confidence interval [CI]) of internalizing problems according to quintiles of household income** | | | | | **Overall prevalence** | **Inequalities in internalizing problems (least deprived ref v most deprived)^d^** | |
| --- | --- | --- | --- | --- | --- | --- | --- |
| 1/lowest | 2 | 3 | 4 | 5/highest | N=5,467 | Risk ratio (CI) | Risk difference, % (CI) |
| **Model A:** Unadjusted^a^ | | | | | | | |
| 16 (13, 20) | 13 (10, 16) | 9 (7, 11) | 8 (6, 10) | 5 (4, 7) | 10 (9, 11) | 3.0 (1.0, 4.0) | 11 (7, 14) |
| **Model B:** Adjusted for baseline confounders/ TDE^b^ | | | | | | | |
| 14 (9, 19) | 11 (8, 13) | 9 (7, 11) | 9 (6, 11) | 8 (3, 12) | 10 (8, 11) | 1.9 (0.9, 2.9) | 6 (1, 11) |
| **Model C:** Observed controlled direct effect/ CDE^c^ | | | | | | | |
| 14 (9, 19) | 11 (9, 14) | 9 (7, 11) | 9 (6, 12) | 7 (4, 11) | 10 (9, 12) | 1.9 (0.9, 3.0) | 7 (1, 12) |
| **Moderate-to-vigorous physical activity interventions simulations** | | | | | | | |
| **Scenario 1: Universal achievement of WHO target (**60min moderate-to-vigorous physical activity per day) | | | | | | | |
| 14 (8, 21) | 11 (7, 15) | 9 (6, 12) | 9 (5, 12) | 7 (3, 11) | 10 (7, 13) | 1.9 (0.9, 3.0) | 7 (1, 12) |
| **Scenario 2: Universal, school break time** (Model C/ CDE, plus average 2.3min increase, 85% uptake | | | | | | | |
| 14 (9, 20) | 11 (9, 14) | 9 (7, 11) | 9 (6, 12) | 7 (4, 11) | 10 (8, 12) | 1.9 (0.9, 3.0) | 7 (1, 12) |
| **Scenario 3: Universal, active transport** (Model C/ CDE, plus average 4min increase for non-active and 2min for active in one direction, 77% uptake | | | | | | | |
| 14 (9, 19) | 11 (9, 14) | 9 (7, 11) | 9 (6, 12) | 7 (4, 11) | 10 (8, 12) | 1.9 (0.9, 3.0) | 7 (1, 12) |
| **Scenario 4: Targeted, after school** (Model C/ CDE, plus 2.6 min increase in lowest quintile of material deprivation, differential uptake^e^ | | | | | | | |
| 14 (9, 20) | 11 (9, 14) | 9 (7, 11) | 9 (6, 12) | 7 (4, 11) | 10 (8, 12) | 1.9 (0.9, 3.0) | 7 (1, 12) |
| **Scenario 5: Indicated** (Model C/ CDE, plus 6.7 min increase for children with high SDQ at 5 years, 64% uptake | | | | | | | |
| 14 (9, 19) | 11 (9, 14) | 9 (7, 11) | 9 (6, 12) | 7 (4, 11) | 10 (8, 12) | 1.9 (0.9, 3.0) | 7 (1, 12) |
| **Scenario 6: Proportionate universal intervention (scenarios 2-5)** | | | | | | | |
| 14 (9, 20) | 11 (9, 14) | 9 (7, 11) | 9 (6, 12) | 7 (4, 11) | 10 (8, 12) | 1.9 (0.9, 3.0) | 7 (1, 12) |

^a^ Weighted for attrition to the physical activity study

^b^ Total direct effect (TDE) of income on internalizing mental health problems adjusted for attrition to physical activity study and baseline confounders

^c^ Observed controlled direct effect (CDE) of income on internalizing mental health problems, adjusted for attrition to physical activity study, baseline confounders, observed moderate-to-vigorous physical activity and time-varying confounders at 7 years

^d^ Relative and absolute inequalities were modelled using a continuous linear term for income, explaining discordance from prevalence estimates

^e^74% uptake below poverty line, 80% uptake for the rest – overall uptake 77%)
